# Designing Superhydrophilic 3D Porous Surfaces on Polyetherketoneketone Surfaces to Promote Biocompatibility

**DOI:** 10.3390/jfb16030106

**Published:** 2025-03-14

**Authors:** Hui-Ching Lin, Chiang-Sang Chen, Kai-Yi Lin, Ya-Lin Huang, Hao-Hsiang Hsu, Yu-Lin Kuo, Wei-Cheng Chen, Her-Hsiung Huang

**Affiliations:** 1 Department of Dentistry, National Yang Ming Chiao Tung University, Taipei 112, Taiwan; das49@tpech.gov.tw (H.-C.L.); ccs0102@femh.org.tw (C.-S.C.); yhuang@nycu.edu.tw (Y.-L.H.); how849901@nycu.edu.tw (H.-H.H.); 2 Department of Dentistry, Taipei City Hospital Renai Branch, Taipei 106, Taiwan; 3 Department of Health and Welfare, University of Taipei, Taipei 100, Taiwan; 4 Department of Orthopedic Surgery, Far Eastern Memorial Hospital, New Taipei City 220, Taiwan; 5 Department of Materials and Textiles, Asia Eastern University of Science and Technology, New Taipei City 220, Taiwan; 6 Institute of Oral Tissue Engineering and Biomaterials, National Yang Ming Chiao Tung University, Taipei 112, Taiwan; ab.eric.de13@nycu.edu.tw; 7 Department of Mechanical Engineering, National Taiwan University of Science and Technology, Taipei 106, Taiwan; ylkuo@mail.ntust.edu.tw; 8 Department of Medicine, MacKay Medical College, New Taipei City 252, Taiwan; 9 Division of Sports Medicine & Surgery, Department of Orthopedic Surgery, MacKay Memorial Hospital, Taipei 104, Taiwan; 10 Department of Medical Research, China Medical University Hospital, China Medical University, Taichung 404, Taiwan; 11 Department of Bioinformatics and Medical Engineering, Asia University, Taichung 413, Taiwan; 12 School of Dentistry, Kaohsiung Medical University, Kaohsiung 807, Taiwan; 13 Department of Education and Research, Taipei City Hospital, Taipei 103, Taiwan

**Keywords:** polyetherketoneketone, implant, surface treatment, biocompatibility

## Abstract

Polyetherketoneketone (PEKK) exhibits satisfactory mechanical properties and biocompatibility, with an elastic modulus closely resembling that of natural bone. This property reduces the stress-shielding effect associated with bone implants. However, the biological inertness of the PEKK surface remains a significant limitation for its application in bone tissue engineering. The objective of this study was to create a superhydrophilic 3D porous structure on the surface of PEKK to enhance biocompatibility, in terms of vascularization and bone remodeling. A combination of mechanical, chemical, and physical surface treatments was employed to modify the PEKK surface. Initially, mechanical sandblasting was used to create a rough surface to promote mechanical interlocking with bone tissue. Subsequently, chemical acid etching and physical low-temperature atmospheric plasma cleaning were applied to develop a superhydrophilic 3D porous surface. The modified surfaces were characterized for morphology, roughness, hydrophilicity, and functional groups. Cellular responses, including vascularization and bone remodeling, were evaluated to assess the potential for improved biocompatibility. The combination of acid etching and low-temperature atmospheric plasma cleaning, with or without prior sandblasting, successfully created a superhydrophilic 3D porous structure on the PEKK surface. This modified surface enhanced the tube formation in human umbilical vein endothelial cells. It also promoted the adhesion and mineralization of human bone marrow mesenchymal stem cells and slightly reduced tartrate-resistant acid phosphatase expression and F-actin ring size in mouse macrophage cells. This study introduces an innovative and effective surface modification strategy for PEKK surface, combining mechanical, chemical, and physical treatments to enhance biocompatibility. The modified PEKK surface promotes angiogenic and osteogenic responses while slightly inhibiting osteoclastic activity, making it a potential alternative for dental and orthopedic PEKK implant applications.

## 1. Introduction

In bone tissue engineering, metallic implants are commonly used as materials for bone implants due to their excellent biocompatibility and mechanical strength. However, the elastic modulus of traditional metallic implants (>100 GPa) is significantly higher than that of human bone (~1–30 GPa), resulting in a mismatch of mechanical properties between the implant and surrounding natural bone. This mismatch often leads to stress-shielding effects around the implant, causing bone resorption and subsequent implant loosening [1]. Additionally, metallic implants may generate metal particles and ions due to fretting corrosion within the human body, which can induce inflammatory responses and activate osteoclasts, further causing bone resorption and related complications [2].

In recent years, polyetherketoneketone (PEKK) has emerged as a potential alternative for metallic dental and orthopedic implants due to its elastic modulus (~4 GPa), which is closer to that of bone tissue. PEKK also offers good biocompatibility, low inflammatory response, and good chemical stability [3,4]. Despite these advantages, the biologically inert surface of PEKK limits its osseointegration potential, which can ultimately lead to implant loosening [5]. Overcoming or improving this limitation represents the greatest challenge for the clinical application of PEKK implants [6]. Furthermore, the hydrophobic surface of PEKK is unfavorable for cell adhesion, thereby delaying osseointegration. In contrast, hydrophilic surfaces facilitate blood wetting, enhance protein adsorption, and can play a role in anti-inflammatory immune modulation, accelerating new bone formation around the implant [7]. Given the aforementioned biological inertness and hydrophobicity of PEKK surfaces, applying appropriate surface treatments to enhance PEKK’s bioactivity and hydrophilicity is a viable approach [5]. However, enhancing the biocompatibility of PEKK surfaces remains a challenging area of research.

Studies have shown that sandblasted implant surfaces possess optimal roughness, which not only enhances stable mechanical interlocking between the implant and bone tissue [8] but also provides a favorable microenvironment for cell adhesion and growth, further promoting osteoblast differentiation [9]. A study indicated that sandblasted PEKK implant surfaces also exhibit micron-scale porous structures [10]. However, sandblasting reduces surface hydrophilicity, which can negatively impact cell adhesion and subsequent osseointegration [11]. Therefore, combining sandblasting with other surface treatment techniques, which can improve the surface hydrophilicity, is necessary to address these limitations. Furthermore, chemical sulfonation is one of the most commonly used surface treatment methods for PEKK [12]. Sulfonation involves immersing PEKK in concentrated sulfuric acid, which etches the PEKK surface, creating nanostructured and microporous features on the surface, thereby increasing surface roughness [13]. Implant surfaces with appropriate porous structures promote osteoblast growth, differentiation, and migration, enabling cells to grow into the pores, while also enhancing vascularization and improving the osseointegration between implants and bone tissue [14]. Beside the abovementioned surface roughness and porosity, surface hydrophilicity is undoubtly critical in biomedical material applications. Research shows that cells adhere more readily to hydrophilic materials, and compared to hydrophobic materials, hydrophilic surfaces improve the spread of cells [15]. In recent years, plasma treatment has been widely utilized to modify material surfaces, changing surface morphology and chemical bonding while introducing hydrophilic functional groups to enhance surface hydrophilicity. Plasma surface treatment has garnered significant attention due to its simplicity, repeatability, cost-effectiveness, energy efficiency, and compatibility with other surface treatment techniques, making it highly promising for further development in implant applications [16].

The synergistic application of the above surface treatment processes shows promising clinical potential for enhancing the biocompatibility of PEKK implants. The objective of this study was to fabricate a superhydrophilic 3D porous PEKK surface by simply combining mechanical sandblasting, chemical sulfonation, and physical low-temperature (<40 °C) atmospheric plasma treatments. This simple and rapid hybrid surface treatment process was expected to positively affect the angiogenesis and bone remodeling of PEKK implant surfaces during osseointegration.

## 2. Materials and Methods

**Figure 1:**
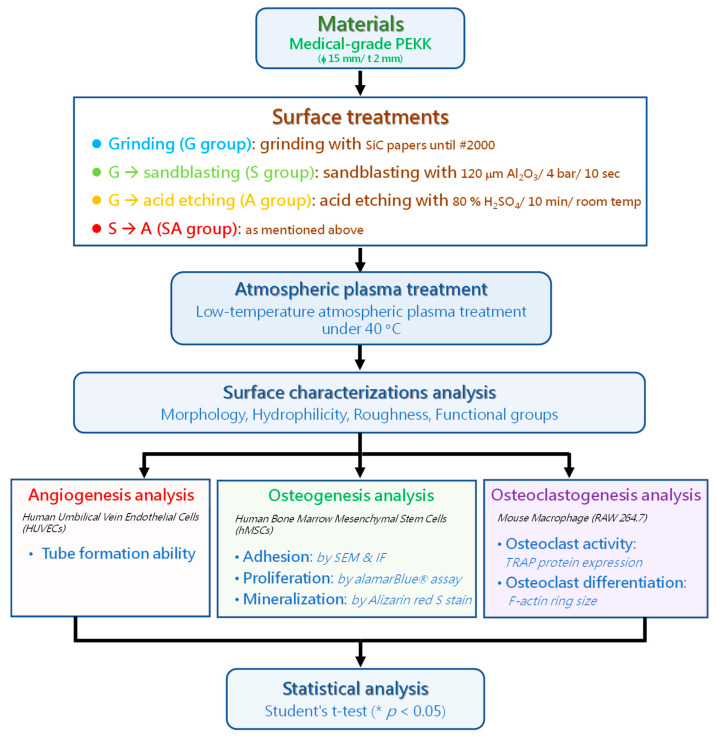
Flow chart of the experimental procedure.

Figure 1 presents the flow chart of the experimental procedure. The corresponding details are explained below.

### 2.1. Materials’ Preparations and Surface Treatments

Medical-grade PEKK discs (Cendres+Métaux SA, Biel, Switzerland) were purchased and digitally machined into disc-shape specimens with a diameter of 15 mm and a thickness of 2 mm. The PEKK discs were sequentially ground with silicon carbide papers until #2000.

For roughening the ground PEKK surface, the ground specimens were mechanically sandblasted using a dental sandblasting machine under the following conditions: 4 bar nozzle pressure, 10 cm nozzle distance, and 120 μm alumina particles for 10 s. After ultrasonic cleaning in 95% ethanol for 5 min, the sandblasted specimens were then air-dried and stored in a desiccator.

For creating a 3D porous surface on the PEKK surface, chemical etching, i.e., sulfonation treatment, was performed for the ground and the sandblasted PEKK surfaces by immersing the specimens in an 80% sulfuric acid solution at room temperature for 10 min. After sequential ultrasonic cleaning in acetone, 95% ethanol, and deionized water for 5 min, the acid-etched specimens were then air-dried and stored in a desiccator.

To enhance surface hydrophilicity, a physical low-temperature atmospheric plasma cleaning treatment using argon as the working gas was applied to the abovementioned four groups, including ground, sandblasted, ground + acid etched, and sandblasted + acid etched groups, with the designations of G (control), S, A, and SA, respectively. The conditions for plasma treatment were as follows: radio frequency (RF) power 500 W with frequency 32 kHz, working gas argon with flow rate 27 standard liters per minute (slm) and pressure 2.74 kg/cm^2^, plasma head-to-specimen surface distance 3 mm, treatment duration 25 s, and specimen surface temperature < 40 °C. Afterwards, the plasma-treated specimens were then stored in a desiccator.

### 2.2. Surface Characterization Analysis

The surface morphologies of the specimens were observed using a field emission scanning electron microscope (FE-SEM) (JSM-6500F, JEOL, Tokyo, Japan) operating at 15 kV after coating with a 10 nm thick conductive Pt film. Hydrophilicity was analyzed using a contact angle goniometer (100SB, Sindatek, New Taipei City, Taiwan) to measure the contact angle of deionized water. Surface roughness parameters (arithmetic mean height (Sa), root mean square height (Sq), and maximum peak-to-valley height (Sz)) were measured using a 3D profilometer (Profilm 3D, KLA-Filmetrics, San Diego, CA, USA). Chemical functional groups on the specimen surfaces were identified using Fourier transform infrared spectroscopy (FTIR) (Vertex 80v, Bruker, Ettlingen, Germany).

### 2.3. Angiogenesis Analysis

#### Tube Formation Ability

Human umbilical vein endothelial cells (HUVECs) which were purchased from the Bioresource Collection and Research Centre (BCRC) in Hsinchu, Taiwan were used. The culture medium was Medium 199 containing 10% fetal bovine serum (FBS), endothelial cell growth supplement, and sodium bicarbonate. Firstly, the HUVEC cells were seeded onto test specimens at a density of 2 × 10⁴ cells/150 μL/disc. After 72 h of cell incubation, we collected the culture medium from each specimen as the condition medium for the following tube formation ability analysis. Concurrently, we coated the bottom of an ibidi^®^ µ-Slide 15 Well 3D culture plate with low growth factor matrix gel (Corning^®^ Matrigel^®^ Matrix) and incubated for 1 h to allow gelation. Sequentially, we mixed HUVECs with the pre-collected condition medium and seed them onto the gel at a density of 1 × 10⁴ cells/50 μL/well. The vascular endothelial growth factor (VEGF)-containing culture medium served as a positive control, as it is expected to promote tube formation. Tube formation was observed and imaged using an optical microscope at 1 and 2.5 h post-seeding. Tube formation ability was assessed by quantifying the number of tubes within the observed field.

### 2.4. Bone Remodeling Analysis

#### 2.4.1. Osteoblastic Response

Human bone marrow mesenchymal stem cells (hMSCs) were used. The cell culture medium was Dulbecco’s modified eagle medium–low glucose (DMEM) containing 10% FBS and sodium bicarbonate. For cell adhesion morphology observation, hMSCs (10^4^ cells/disc) were cultured on specimens for 3 h. After fixation and sequential dehydration, FE-SEM was used to observe cell adhesion morphology following Pt coating for conductivity. For adhesion-related protein expression, vinculin (focal adhesion complex) and F-actin (cytoskeletal arrangement) were analyzed after 3 h of culture using a fluorescence microscope (AZ100, Nikon, Tokyo, Japan) with Image-Pro Express 6.0 (Media Cybernetics, Rockville, MD, USA). For the cell proliferation assay, hMSCs (10^4^ cells/disc) were cultured on specimens, and proliferation over 5 days was assessed using the alamarBlue^®^ assay. Cell viability was determined by the percentage reduction in alamarBlue^®^ reagent, where a higher reduction indicated greater cell proliferation. For cell mineralization assay, hMSCs (10⁴ cells/disc) were cultured on specimens for 14 days, and mineralization was evaluated using the Alizarin Red S staining assay. Mineral deposition was qualitatively assessed via optical microscopy (BX51M, Olympus, Tokyo, Japan), with increased red staining indicating higher mineralization. Quantitative analysis was performed by measuring optical density (OD) at 540 nm using a microplate reader (Multiskan FC, Thermo Scientific, Vantaa, Finland), where higher OD values corresponded to greater mineralization.

#### 2.4.2. Osteoclastic Activity

RAW 264.7 mouse macrophage cells (2 × 10⁴ cells/disc) were cultured in DMEM with 10% FBS and sodium bicarbonate for 24 h on specimen surfaces. Cells were then treated with 50 ng/mL RANKL-induced osteoclastic differentiation medium, refreshed every 50–60 h. After 1 and 5 d, cells were fixed with 10% formalin, permeabilized with 0.3% Triton X-100, and blocked with 5% normal goat serum. Primary antibody (anti-tartrate-resistant acid phosphatase, anti-TRAP) was applied at 4 °C for 20 h, followed by Alexa Fluor^®^ 488 secondary antibody for 1.5 h. Cytoskeletal F-actin was stained with rhodamine phalloidin for 1 h, and nuclei were stained with DAPI for 15 min. Fluorescence images, taken by a fluorescent microscope (AZ100, Nikon, Tokyo, Japan), were analyzed, where increased green TRAP signals and larger red F-actin rings indicated higher osteoclastic activity.

### 2.5. Statistical Methods

Each type of experiment was conducted independently on three separate occasions. During each of these occasions, three individual samples were tested, ensuring a total of nine samples per experiment type. The data are reported as mean ± standard deviation (SD). The statistical significance between groups was determined using a Student’s *t*-test (G group as control). A *p* value < 0.05 was considered statistically significant.

## 3. Results

### 3.1. Surface Characterization

**Figure 2:**
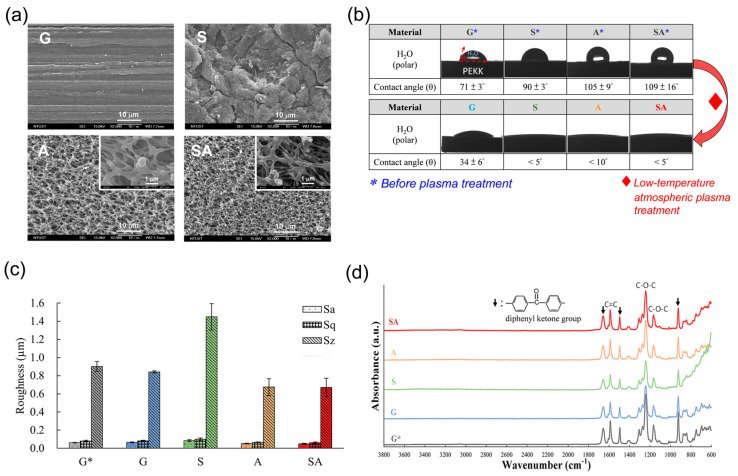
Surface characterizations of the test specimens: (**a**) morphology; (**b**) hydrophilicity; (**c**) roughness; (**d**) functional groups.

Figure 2 presents the surface characterizations of the test specimens: (a) morphology; (b) hydrophilicity; (c) roughness; (d) functional groups. For surface morphology (Figure 2a), the four different test groups (G, S, A, and SA) varied distinctly. The G surface exhibited parallel grooves produced during the grinding process, while the sandblasted S surface presented a mixed convex/concave morphology. Both the A and SA surfaces displayed 3D interconnected pores, with the pore size being less than 1.5 μm. For surface hydrophilicity (or water contact angle) (Figure 2b), the low-temperature atmospheric plasma treatment had a pronounced effect on the hydrophilicity of the specimens. The surfaces of test four groups before plasma treatment (i.e., G*, S*, A*, and SA*) exhibited hydrophobic characteristics, with water contact angles ranging between 70° and 110°, indicative of poor hydrophilicity. However, following the low-temperature atmospheric plasma treatment, the S, A, and SA surfaces underwent a significant transformation, becoming superhydrophilic with water contact angles dropping to less than 10°. Figure 2c,d present the surface roughness and functional groups of the test specimens, respectively. All test specimens revealed similar surface roughness and functional groups, except the S group, which had a higher surface roughness Sz. Note that the low-temperature atmospheric plasma treatment had no significant effect on surface morphology, roughness, or functional groups.

### 3.2. Angiogenesis

**Figure 3:**
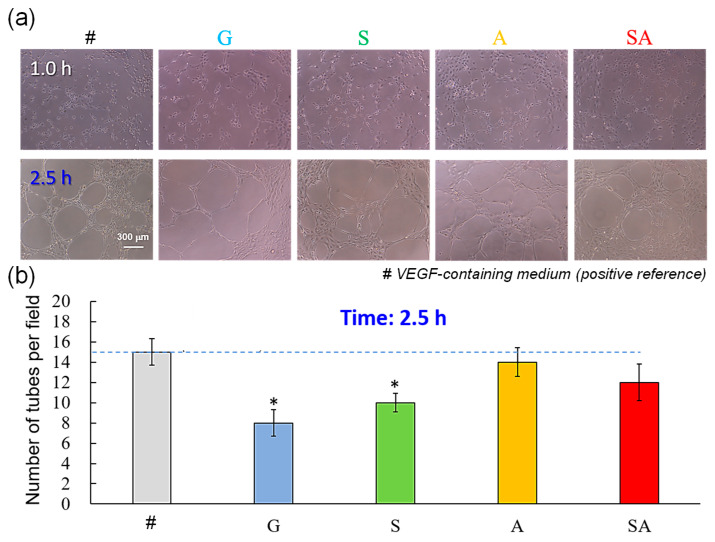
Angiogenesis property, in terms of tube formation ability, by HUVECs exposed to the condition medium of the test specimens: (**a**) optical microscope micrograph at 1 and 2.5 h; (**b**) corresponding number of tubes per field at 2.5 h (# VEGF-containing medium (positive reference); * *p* < 0.05 vs. VEGF-containing medium).

Figure 3 presents the angiogenesis property, in terms of tube formation ability, by HUVECs exposed to the condition medium of the test specimens: (a) optical microscope micrograph at 1 and 2.5 h; (b) corresponding number of tubes per field at 2.5 h. The VEGF-containing medium was used as a positive reference. As shown in Figure 3b, the test specimens demonstrated varying levels of tube formation ability, with the A group showing the highest ability (1.7-fold increase in tube formation compared to the G group), followed by the SA group (1.5-fold), S group (1.2-fold), and G group (control), which exhibited the least ability to induce tube formation. Although the VEGF-containing medium showed the best tube formation ability, the A and SA groups also showed compatible tube formation ability with the positive reference (VEGF-containing medium).

### 3.3. Osteogenesis

**Figure 4:**
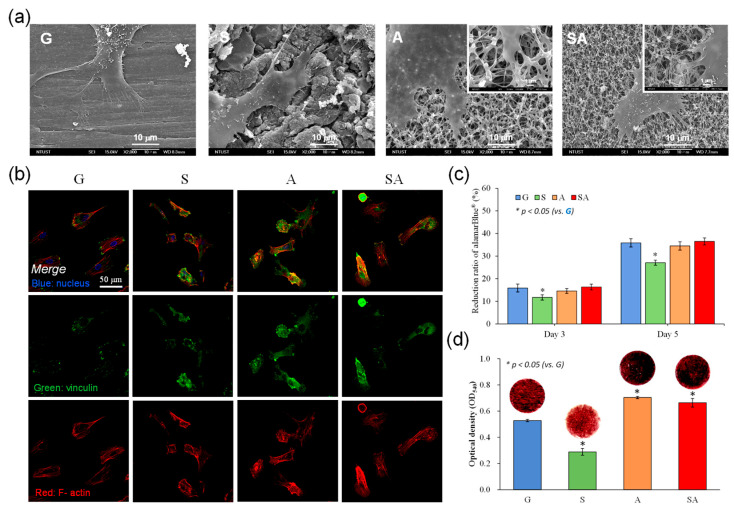
Osteogenesis property, in terms of adhesion, proliferation, and mineralization, by hMSCs cultured on the test specimens: (**a**) 3 h cell adhesion morphology observed by FE-SEM; (**b**) 3 h cell adhesion morphology observed by immunofluorescent staining; (**c**) 5 d cell proliferation analyzed by alamarBlue® assay; (**d**) 14 d cell mineralization analyzed using Alizarin red S staining (* *p* < 0.05 vs. G).

Figure 4 presents the osteogenesis property, in terms of adhesion, proliferation, and mineralization, by hMSCs cultured on the test specimens: (a) 3 h cell adhesion morphology observed by FE-SEM; (b) 3 h cell adhesion morphology observed by immunofluorescent staining; (c) 5 d cell proliferation analyzed by alamarBlue® assay; (d) 14 d cell mineralization analyzed using Alizarin red S staining. In terms of cell adhesion, cells in the S and SA groups demonstrated pronounced cellular filopodia (Figure 4a), which extended into the 3D porous structure, indicating an enhanced interaction with the surface features. However, the expression of adhesion-related protein, such as vinculin, and the cytoskeletal arrangement, indicated by F-actin distribution, were observed to be least significant on S group (Figure 4b). As shown in Figure 4c, cellular proliferation followed the order A~SA~G > S. This trend indicated that both the A and SA groups supported comparable cell proliferation when compared to the G group, with the S group demonstrating the lowest cellular proliferation capacity. As shown in Figure 4d, the mineralization process, assessed by the deposition of mineralized matrix, followed the order A~SA > G > S. Both the A and SA groups facilitated superior mineralization compared to the G group (control), with S showing the lowest mineralization potential.

### 3.4. Osteoclast Genesis

**Figure 5:**
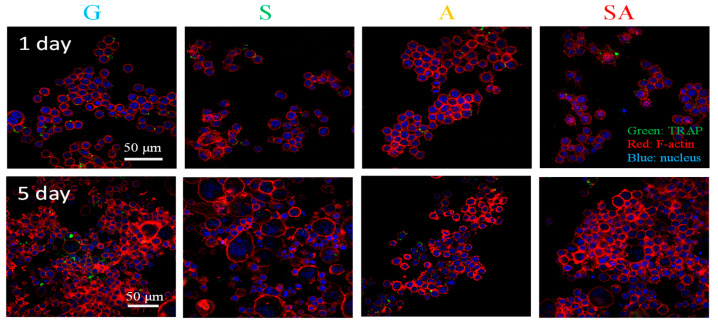
Osteoclastogenesis property, in terms of TRAP expression (green point) and F-actin ring size (red color) observed by immunofluorescent staining, by RAW 264.7 macrophage cells cultured on the test specimens for 1 and 5 d.

Figure 5 presents the osteoclastogenesis property, in terms of TRAP expression (green point) and F-actin ring size (red color) observed by immunofluorescent staining, by RAW 264.7 macrophage cells cultured on the test specimens for 1 and 5 d. At day 1, there were no significant differences in TRAP protein expression and F-actin ring size between the test specimens. At day 5, the osteoclast activity, as determined by TRAP protein expression, demonstrated a rank order of A ≈ SA ≈ S < G, where specimens A, SA, and S exhibited comparable TRAP levels, which were slightly lower than those observed in the G specimen. For osteoclast differentiation, evaluated by the size of the F-actin ring the A, SA, and G specimens exhibited comparable F-actin ring sizes, with a reduction from approximately 0.3- to 0.5-fold in size compared to the S specimen. Overall, the A and SA groups exhibited a slight inhibition in osteoclastogenesis compared to the G group (control).

## 4. Discussion

### 4.1. Surface Characterization

#### 4.1.1. Roughness

The PEEK manufactured via fused filament fabrication, characterized by high surface roughness, exhibits a significantly increased osteoblast proliferation and metabolic activity compared to smooth PEEK treated with grit-blasting [17]. Surface modifications, such as sandblasting, of PEEK at the micrometer scale have been employed to improve the behavior of MG-63 cells and enhance bone-bonding capability [18]. Nevertheless, an early report found that excessive surface roughness (Ra > 2.19 μm) can hinder osteoblast adhesion due to challenges in forming osteoblastic pseudopodia between larger crests and grooves [19]. The values of surface roughness of the test four groups in Figure 2c were well below the abovementioned 2.19 μm and thus are expected to be suitable for osteoblastic adhesion, as shown in Figure 4a,b. Furthermore, the S group with the highest roughness Sz 1.4 μm would relatively provide a better mechanical interlocking stability between the surface and surrounding bone.

The sandblasting and acid etching (SLA) process is considered one of the gold standards for titanium dental implant surface treatment. For commercially available SLA-treated titanium dental implants, some surface roughness parameters have been reported as follows: Sa values typically range from 0.9 to 1.5 µm, and Sz values span from 6.0 to 12.0 µm [20]. In a previous in vivo study, it was demonstrated that SLA-treated titanium dental implants with surface roughness values of approximately Sa = 1.0 µm and Sz = 9.0 µm exhibit successful osseointegration [21]. In contrast, the surface roughness values obtained in the current study (Figure 2c) were notably lower than those reported in the abovementioned literature. Consequently, further investigation into increasing the surface roughness of the proposed PEKK may enhance osseointegration in vivo, thereby offering potential clinical applications.

#### 4.1.2. Porosity

Emulating the intricate morphology of trabecular bone plays a pivotal role in the design of porous implant surfaces. Compared to conventional rough surfaces, porous PEEK surfaces have demonstrated superior osteoblastic differentiation and enhanced bonding strength with bone tissue [22]. Various strategies have been employed to create porous structures on PEEK, including sulfonation, melt extrusion, porogen templating, and ion implantation [23]. Among these, sulfonation not only produces a three-dimensional porous network but also introduces -SO_3_H functional groups, which contribute to pre-osteoblast activity, apatite deposition, and bone growth [23]. Furthermore, due to its higher ketone content, PEKK undergoes sulfonation more effectively than PEEK, resulting in more micropores and -SO_3_H groups [24]. The sulfonation procedure of PEEK increases surface porosity and roughness, leading to osteoblastic cell adhesion, proliferation, and differentiation [25]. The optimal sulfonation conditions of PEEK and PEKK include acid concentration, temperature, and treatment duration, etc., which help to achieve the desired surface porosity for the improvement in cellular responses [26,27].

Sulfonation treatment induces the formation of a porous structure on both PEEK and PEKK, with pore sizes typically ranging from a few micrometers to approximately 10 µm and a porous layer thickness varying from a few micrometers to several tens of micrometers [13,28,29]. The final pore characteristics depend on multiple factors, including sulfuric acid concentration, immersion duration, and polymer composition. As observed in Figure 2a, the sulfonation process employed in this study resulted in a smaller pore size (<1.5 µm) and a thinner porous layer (estimated below 10 µm) compared to previously reported values. Given these characteristics, the influence of the porous morphology on the bulk mechanical properties of PEKK in clinical applications is expected to be negligible.

In this study, we applied a simple and rapid sulfuric acid-etching treatment to create a 3D porous structure on both ground and sandblasted PEKK surfaces (Figure 2a), i.e., the A and SA groups, respectively. This 3D porous scaffold structure, similar to the extracellular matrix (ECM), is expected to support the cellular attachment, migration, and organization [30]. To further reinforce the biocompatibility of porous PEKK, a promising superhydrophilic surface was created by low-temperature atmospheric plasma treatment on the porous PEKK surface produced by sulfonation process.

#### 4.1.3. Hydrophilicity

The dramatic reduction in the contact angle (<10 °C) of the S, A, and SA groups, shown in Figure 2b, suggested that the low-temperature atmospheric plasma treatment significantly induced hydrophilicity. Notably, the G group did not significantly decrease the water contact angle following the plasma treatment, which may be attributed to its distinct surface character.

Note that the application of the low-temperature atmospheric plasma treatment did not induce any significant changes in the surface morphology and roughness of the four test groups. This suggests that the low-temperature atmospheric plasma treatment conditions, though increasing the surface hydrophilicity (Figure 2b), did not significantly modify the fundamental topographical characteristics of these specimens. Any variations observed in cell responses between the different test groups are more likely to be related to differences in hydrophilicity and morphology, as will be discussed later.

The literature suggests that the porous structure formed by sulfonation on hydrophobic PEEK does not notably enhance its surface hydrophilicity [31] and may even reduce it [29,32], despite the presence of hydrophilic -SO₃H groups. However, oxygen plasma treatment significantly increases the hydrophilicity of the sulfonated PEEK surface with a water contact angle of approximately 45° [29]. In contrast, this study demonstrated that a straightforward low-temperature atmospheric plasma treatment effectively transformed the hydrophobic sulfonated PEKK surface into a superhydrophilic one with a water contact angle < 10°.

The low-temperature atmospheric plasma treatment in this study did not alter the surface morphology or the roughness of PEKK but did induce surface superhydrophilicity (Figure 2). Given the parameters used for the plasma treatment, we anticipated that ion bombardment would introduce polar functional groups, remove organic contaminants, and generate temporary surface charges. This combined effect was expected to enhance surface hydrophilicity.

The polar functional groups expected to form on the PEKK surface during the plasma treatment, such as –OH, –COOH, and –C=O, significantly overlap with the intrinsic functional groups of PEKK. Moreover, their concentration may be relatively low, making it challenging to distinctly detect them in the FTIR spectra (Figure 2d). Furthermore, during the plasma treatment, energetic species (e.g., ions and electrons) interact with the polymer surface, leading to charge deposition. Since PEKK is a poor conductor, the deposited charge cannot easily dissipate and remains on the surface temporarily. This phenomenon is often referred to as surface charge accumulation, which can induce dipole formation or enhance surface energy, leading to an increase in hydrophilicity. Therefore, surface potential decay measurements can be utilized in future studies to examine the impact of charge-related effects on the hydrophilicity of PEKK following plasma treatment.

Generally, the glass transition temperature (Tg) of PEKK typically ranges between 160 °C and 180 °C, and its melting point is generally from around 340 °C to 360 °C, depending on the specific grade and formulation of the material. In this study, during the low-temperature atmospheric plasma treatment, the surface temperature of the specimen remained below 40 °C (ranging from 34 °C to 36 °C), as measured by a precise thermocouple. The treatment duration for each specimen was 25 s. Under these conditions, we expected that the short duration of the low-temperature atmospheric plasma treatment would not affect the crystallinity of PEKK, and thus, the heating effect on crystallinity was considered negligible in this study.

In the current study, the low-temperature (<40 °C) atmospheric plasma treatment significantly improved the hydrophilicity of the S, A, and SA groups, which was consistent with previous findings that traditional plasma treatments can enhance the wettability of the hydrophobic PEEK surface, leading to the improvement in cell responses [33,34,35]. The low-temperature atmospheric plasma utilized in this study is anticipated to demonstrate greater potential in biomedical applications compared to conventional high-temperature plasma, primarily due to its low operational temperature and the atmospheric conditions maintained during treatment.

### 4.2. Angiogenesis

The ability to induce endothelial tube formation in vitro is a key indicator of angiogenic potential, reflecting the ability of endothelial cells to migrate, proliferate, and organize into capillary-like structures. The results in Figure 3 suggest that the A group had the highest angiogenic potential, as evidenced by a 1.7-fold increase in tube formation ability when compared to that of the control (G group). The SA group, which demonstrated a 1.5-fold increase in tube formation ability, also appeared to enhance angiogenic response, though to a little lower degree than the A group. This indicates that the superhydrophilic 3D porous surface on PEKK, with or without sandblasting treatment, enhanced endothelial cell behavior. In contrast, the G group, serving as the control, exhibited minimal or no enhancement in tube formation ability, thus reinforcing that the experimental specimens, particularly the A and SA groups, possessed distinct pro-angiogenic effects.

Angiogenesis is greatly important as it increases the transportation of oxygen and nutrients needed for the bone healing and reconstruction process [36]. VEGF significantly promotes the proliferation, migration, and angiogenesis of endothelial cells [37]. Therefore, the VEGF-containing medium was used as a positive reference. A recent study has shown the hierarchical microstructure of ECM functions as an angiogenic biomaterial [38]. This may partially explain the fact the condition medium of the S or SA group with a 3D porous surface feature, similar to hierarchical microstructure of the ECM, showed good, comparable tube formation ability to that of the VEGF-containing medium group, although the VEGF-containing medium group still had the best tube formation ability. Additionally, the hydrophilic modification of the PEEK surface significantly enhances the tube formation ability of HUVECs [35]. Thus, the superhydrophilic character of the S and SA surfaces also play a positive role in enhancing the tube formation ability in HUVEC cells. However, the further enhancement in angiogenesis on the S and SA surfaces remains a subject of concern for future research.

### 4.3. Osteogenesis

As shown in Figure 4, the superhydrophilic 3D porous structure on PEKK surfaces, including the A and SA groups, had a better adhesion, proliferation, and mineralization of the ECM in hMSCs, particularly adhesion and mineralization ability, among the test groups. Previous studies have confirmed that the enhancement in the hydrophilicity on PEEK surface leads to the improvement in cell responses [33,34]. The microporous feature on PEEK surface improves the osteoblastic cell adhesion and proliferation [35]. Additionally, the hydrophilic modification significantly enhances the interaction between the PEEK surface and cells, including the mineralization of the ECM of osteoblastic cells [35].

Therefore, as shown in Figure 4, the observed differences in cell adhesion, proliferation, and mineralization across the four test groups may be explained by a combination of surface features, including morphology and hydrophilicity, both of which influence cell behavior. The presence of the superhydrophilic 3D porous feature of the A and SA groups likely promoted enhanced cellular filopodia extension, providing a greater contact area for adhesion. Sequentially, this unique surface feature on the A and SA groups supported the subsequent cell growth and enhanced mineralization of the ECM in hMSCs.

### 4.4. Osteoclastogenesis

TRAP is a key marker for osteoclast activity, as it is involved in the resorption of bone and is typically upregulated in mature osteoclasts. In the context of osteoclastogenesis, a higher TRAP expression correlates with an enhanced osteoclastic function, including bone resorption [39]. The slightly decreased TRAP expression in RAW 264.7 cells in the A and SA groups compared to the G group at day 5 in Figure 5 suggests that the superhydrophilic 3D porous structure slightly inhibited the osteoclast activity during the bone remodeling process.

Osteoclasts are responsible for bone matrix resorption and maintaining bone and calcium homeostasis. The F-actin ring, a specialized cytoskeletal structure, is crucial for their bone-resorbing activity [40]. Larger F-actin rings are typically associated with more mature and differentiated osteoclasts. The reduced F-actin ring size in the A and SA groups at day 5 in Figure 5 suggests a slight inhibition in osteoclast differentiation (vs. the G group), whereas the S group showed a more robust osteoclast differentiation, as indicated by the larger F-actin rings. This could indicate that the superhydrophilic 3D porous structure slightly inhibited osteoclast differentiation.

A previous study has provided a detailed understanding of the molecular mechanisms regulating osteoclast activity and differentiation in RAW 264.7 cells, particularly through the analysis of TRAP expression and F-actin ring formation, suggesting an important role for cytolinker protein plectin in osteoclast differentiation and F-actin ring formation through Src binding [33]. However, the mechanism by which the superhydrophilic 3D porous structure in the A and SA groups slightly inhibited osteoclastogenesis in RAW 264.7 cells (vs. the control G group), as shown at day 5 in Figure 5, is interesting and requires further investigations.

### 4.5. Limitations of This Study

This study provided a simple and rapid surface treatment for PEKK surfaces for dental and orthopedic implant applications. The developed superhydrophilic 3D porous PEKK surface enhanced the tube formation of HUVECs and the adhesion and mineralization of hMSCs while slightly inhibiting the TRAP protein expression and F-actin ring size of RAW 264.7 cells. However, the limitations of this study include the lack of in vivo animal experiments to validate the osseointegration potential of the modified PEKK surfaces and the absence of co-culture studies to investigate the interactions between osteoblasts and osteoclasts during bone remodeling. Additionally, the long-term stability and clinical performance of the surface modifications remain to be evaluated. The above important issues are suggested to be investigated in the coming future before the developed PEKK implant surface system is ready for clinical trials.

Additionally, PEKK’s radiolucency allows it to be used in various imaging techniques, including X-rays, computed tomography (CT) scans, and magnetic resonance imaging (MRI) scans, without introducing artifacts or distortions. While research on the luminescent properties of biocompatible calcium phosphates has recently emerged [41], there is currently a lack of specific studies on the potential for photoluminescence-based diagnostics or imaging applications involving biocompatible PEKK. Beyond its potential as a bone implant, as examined in this study, PEKK may offer additional clinical applications, particularly in photoluminescence-based imaging and diagnostics, warranting further research.

The presence of -SO₃H groups on sulfonated PEEK surfaces is commonly identified using FTIR analysis [31,32]. However, a study has also reported the absence of detectable -SO₃H groups on sulfonated PEEK [30], aligning with the findings of this study. Escobar et al. [42] highlighted that a high concentration of -SO₃H groups in sulfuric acid disrupts the chain conformation of PEEK, leading to surface layer swelling. When rinsed with acetone, the swollen PEEK undergoes solidification, during which residual -SO₃H groups dissipate, ultimately forming a 3D porous structure. This mechanism may help explain why -SO₃H groups were not observed on the sulfonated PEKK surface (Figure 2(d)). Another possible explanation is that the quantity of -SO₃H groups in this study was too low for FTIR detection. Furthermore, sulfur atoms on the surface were not detected using energy dispersive X-ray (EDX) analysis, which can be attributed to the reasons mentioned above. To precisely analyze the presence of sulfur on the sulfonated PEKK surface, future studies should consider utilizing X-ray photoelectron spectroscopy (XPS). However, the successful formation of a 3D porous structure on the sulfonated PEKK surface confirms that sulfonation occurred.

## 5. Conclusions

A combination of surface treatments, either acid etching with low-temperature atmospheric plasma cleaning (S group) or sandblasting followed by acid etching and low-temperature atmospheric plasma cleaning (SA group), was employed to create a superhydrophilic 3D porous structure on bioinert PEKK surfaces. This surface modification approach proved effective in promoting the tube formation of HUVECs and the adhesion and mineralization of hMSCs, while also slightly reducing the TRAP protein expression and F-actin ring size of RAW 264.7 cells. These findings present a promising method for enhancing the biocompatibility of PEKK surfaces in dental and orthopedic applications. Future research should also focus on further enhancing the inhibition of osteoclastogenesis and elucidating the molecular mechanisms governing bone remodeling through co-culture studies of osteoblasts and osteoclasts, as well as conducting in vivo animal tests to validate osseointegration.

## Data Availability

The original contributions presented in the study are included in the article, further inquiries can be directed to the corresponding authors.
